# Bayesian geostatistical modelling of stunting in Rwanda: risk factors and spatially explicit residual stunting burden

**DOI:** 10.1186/s12889-022-12552-y

**Published:** 2022-01-24

**Authors:** Vestine Uwiringiyimana, Frank Osei, Sherif Amer, Antonie Veldkamp

**Affiliations:** 1 Faculty of Geo-Information Science and Earth Observation (ITC), University of Twente, P.O. Box 217, 7500 AE Enschede, The Netherlands; 2 Department of Food Science and Technology, College of Agriculture Animal Science and Veterinary Medicine, University of Rwanda, PO Box 3900, Kigali, Rwanda

**Keywords:** Stunting, Spatial pattern, Bayesian modelling, Spatial residuals, Rwanda

## Abstract

**Background:**

Stunting remains a significant public health issue in Rwanda and its prevalence exhibits considerable geographical variation. We apply Bayesian geostatistical modelling to study the spatial pattern of stunting in children less than five years considering anthropometric, socioeconomic and demographic risk factors in Rwanda. In addition, we predict the spatial residuals effects to quantify the burden of stunting not accounted for by our geostatistical model.

**Methods:**

We used the data from the 2015 Rwanda Demographic and Health Survey. We fitted two spatial logistic models with similar structures, only differentiated by the inclusion or exclusion of spatially structured random effects.

**Results:**

The risk factors of stunting identified in the geostatistical model were being male (OR = 1.32, 95% CI: 1.16, 1.47), lower birthweight (kg) (OR = 0.96, 95% CI: 0.95, 0.97), non-exclusive breastfeeding (OR = 1.24, 95% CI: 1.04, 1.45), occurrence of diarrhoea in the last two weeks (OR = 1.18, 95% CI: 1.02, 1.37), a lower proportion of mothers with overweight (BMI ≥ 25) (OR = 0.82, 95% CI: 0.71, 0.95), a higher proportion of mothers with no or only primary education (OR = 1.14, 95% CI: 0.99, 1.36). Also, a higher probability of living in a house with poor flooring material (OR = 1.22, 95% CI: 1.06, 1.41), reliance on a non-improved water source (OR = 1.13, 95% CI: 1.00, 1.27), and a low wealth index were identified as risk factors of stunting. Mapping of the spatial residuals effects showed that, in particular, the Northern and Western regions, followed by the Southern region of Rwanda, still exhibit a higher risk of stunting even after accounting for all the covariates in the spatial model.

**Conclusions:**

Further studies are needed to identify the still unknown spatially explicit factors associated with higher risk of stunting. Finally, given the spatial heterogeneity of stunting, interventions to reduce stunting should be geographically targeted.

## Background

Stunting still is a major public health issue in developing countries. Stunting is an indicator of chronic malnutrition in children less than five years. It is defined as height-for-age that is less than two standard deviations of the World Health Organisation (WHO) child growth standards median [1]. It is the most prevalent form of malnutrition in the world, with globally 149 million children under five stunted [2]. The growth retardation starts during pregnancy and continues until a child is two years of age [3]. Stunting develops from poor maternal health and nutrition, inadequate infant feeding practices, and recurrent and subclinical infections [1]. Stunting is a multifactorial and complex health problem [4] and its direct causes are embedded in the complexity of household, societal, and community factors [5]. The consequences of stunting are felt not only in childhood but also in adulthood. In children, stunting results in decreased motor and cognitive development, impaired immunity, and low education attainment [6]. In adulthood, it leads to lower economic productivity, increases the risk of chronic diseases, and lowers offspring birth weight [6, 7].

Reducing stunting in children under five years of age is among the targets of the Sustainable Development Goals 2 [8]. The aim of Goal 2 is to end all forms of malnutrition by 2030, and by 2025 achieve a 40% reduction in the global prevalence of stunting as set by the World Health Assembly [1]. On a global and regional scale, stunting prevalence varies spatially and temporally. The mapping of child growth failure in Africa [9] showed that, although stunting has reduced overall, there are persistent heterogeneities in levels and trends in stunting across the African continent. Also, effective targeting of interventions to the most vulnerable has been identified as one of the drawbacks in achieving desired targets in stunting reduction. [9] estimated that if interventions and programmes to reduce stunting are not spatially targeted and monitored, there will likely be no African country that will achieve the global nutrition targets in all of its sub-national territories. National stunting prevalence can show improvement, while stunting levels at the local level remain high. In other words, national-level prevalence estimates can mask disparities and inequalities that exist between regions [9]. This is problematic because it is the aggregated prevalence level that is usually used to direct locally implemented interventions and programs for stunting alleviation. Thus, scarce health resources may not be well targeted to the most vulnerable. Also, most studies on the modelling of stunting and identification of its risk factors do not take into account the spatial dependency that exists in the data. Thus, to achieve the set target to reduce stunting, the spatial heterogeneity in stunting prevalence calls for a more in-depth assessment of the drivers of stunting on a local level. Moreover, spatially targeted interventions and programs are required for the most vulnerable communities to benefit from them.

In Rwanda, stunting reduction has been made a priority and this resulted in the national prevalence levels being on the decline in past years. However, with a prevalence of 38% [10], the stunting levels in the country are considered very high according to thresholds of the World Health Organization (WHO) [11]. Also, the disparity in stunting levels at the sub-national level is very apparent, with levels that vary from 59% in some districts to 17% in others [10]. Spatial heterogeneity might be even more pronounced at finer geographical scales. Thus, in this study, we apply Bayesian geostatistical methods to model the spatial patterns of stunting in Rwanda. The model is finally used to make fine scale predictions on 5 × 5 km grid. The use of Bayesian estimation has an important advantage compared to classical statistical modelling approaches due to its capabilities to incorporate prior knowledge about unknown spatial and non-spatial parameters to be estimated. Previous geostatistical analyses done on stunting in Rwanda focused on predicting stunting on a national level using geospatial covariates that are correlated with stunting [9, 12], but did not include child-related factors. Not including child-related covariates in the geostatistical model was signalled as a limitation in these studies. In this study, the aim is to specify a Bayesian geostatistical model using child-related covariates to model stunting and study its risk factors in Rwanda. Also, we aimed to predict the spatial residuals effects to quantify the burden of stunting not accounted for by the specified geostatistical model. The findings of this study are expected to contribute to understanding the spatial heterogeneity of stunting in Rwanda, the risk factors that underline it and will provide new insights into the persistently high levels of stunting in some regions.

## Methods

### Study area

**Fig. 1:**
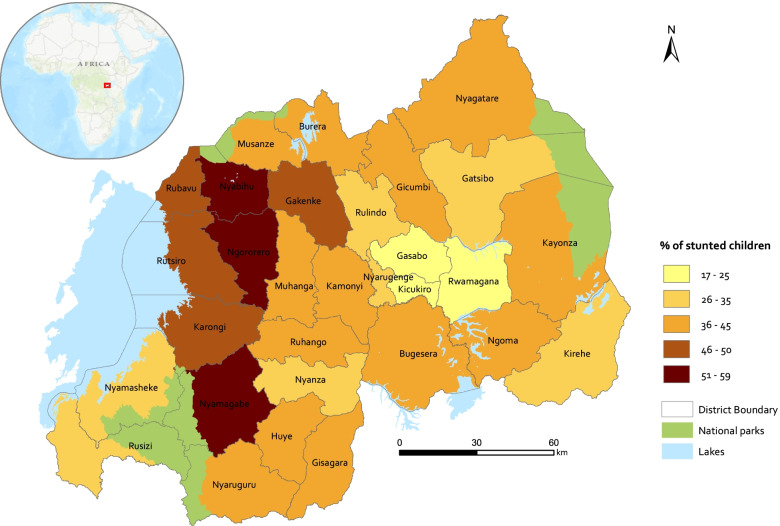
Stunting prevalence per district in Rwanda in 2015 (Source: DHS, 2015). Map created using ArcGIS Desktop version 10.6, licensed (https://www.esri.com/)

Rwanda is located in East African between 1° 04’ and 2° 51’ latitude South, and 28° 50’ and 30° 50’ longitude East. The country’s surface area is 26.338 km^2^ and is bordered by Uganda in the North, Tanzania in the East, Congo in the West, and Burundi in the South. The country’s topography is made up of hills and valleys with the highest point in the country being at 4500 m and the lowest at 980 m. The country is divided into 30 administrative units called districts. The stunting pattern in the country varies spatially with the Western province, located on the Congo Nile trail, being among the most affected, while the central region including Kigali has the least stunting prevalence (Fig. 1).

### Data description

This study was based on the data from the 2015 Rwanda Demographic and Health Survey [10]. In the first stage, clusters within enumeration areas that represent the 30 districts of Rwanda were selected. Clusters are the primary sampling units and they contain on average 100 and 300 households from which 20 to 30 households are randomly selected for survey participation. In Rwanda, a total of 492 clusters were selected, 113 were located in urban areas, and 379 were located in rural areas. The second stage involved the systematic sampling of 26 households within each cluster, making up a sample of 12,792 households on the national level. From a sub-sample consisting of 50% of the households, indices of anthropometric status (child height and weight), individual mother, and household characteristics were recorded for 3,813 children less than five years. The sample size of children less than five years with valid anthropometric data was 3,593 and this study is based on this sample.

### Child-related factors

The Demographic and Health Survey (DHS) data used in this study consisted of anthropometric, demographic, nutritional, and socioeconomic data. The dependent variable was the stunting status of children less than five years, recorded as a dichotomous variable. The explanatory variables were age of the child, birth weight, gender, preceding birth interval, exclusive breastfeeding in the previous six months, use of drugs for intestinal parasites, mothers’ highest education level, and mothers’ BMI. Exclusive breastfeeding and minimum dietary diversity were only measured for children less than two years [10]. The socio-economic variables included the source of drinking water of the household, type of sanitation facility, type of cooking fuels, wealth index, and type of residence. These variables were considered because of their known relation with stunting [5, 13].

### Statistical analysis

Descriptive statistics were conducted using SPSS version 24. Stunting was coded as 0 for non-stunting and 1 for stunting. For the categorical explanatory variables, the value with the least risk of stunting was set as the reference value [14]. Correlation between variables was run to check for multicollinearity with $r> 0.7$. All variables had $r< 0.7$, indicating that multicollinearity did not occur [15]. By taking into account the complex sample design of the DHS survey data, crosstab analysis was conducted on categorical variables with stunting as the dependent variable. For the continuous variables, univariate logistic regression was run to study the association of the variables with stunting. Unadjusted odds ratios were reported for both categorical and continuous variables and a *p*-value of $< 0.05$ was adopted to indicate statistical significance.

For spatial analysis, we conducted the analysis at the individual level, by taking into account the spatial location of household clusters. We fitted a binary logistic model to the Bernoulli outcomes ${y}_{ij}$ defining ${y}_{ij}=1$ if the child $i$ is stunted and ${y}_{ij}=0$ if the child is non-stunted within the $jth$ cluster, $j=1,\dots ,J$ clusters*.* The logistic regression model with the expected probability of becoming stunted being *p* is thus,

$${y}_{ij}\sim Bern \left({p}_{ij}\right)$$

$$\mathrm{logit }\left({p}_{\mathit{ij}}\right)={\beta }_{0}+ \sum_{q=1}^{Q}{\beta }_{q}{X}_{iq} +\sum_{l=1}^{L}{\gamma }_{l}{Z}_{il}+ {s}_{j}+ {u}_{j}$$

where ${\beta }_{0}$ is the intercept, ${\beta }_{1}\dots ., {\beta }_{Q}$ and ${\gamma }_{1}\dots ., {\gamma }_{l}$ are the unknown regression coefficients; ${X}_{iq}$ is a set of continuous covariates (e.g. age, birth weight. See Table 1) and ${Z}_{il}$ is a set of categorical covariates (e.g. sex, exclusive breastfeeding, education, See Table 1); ${s}_{j}$ and ${u}_{j}$ are random effects that allow for spatially structured variation (spatial random effect) and unstructured heterogeneity (non-spatial random effect), respectively.

**Table 1:** Descriptive characteristics of the study population (*n* = 3593)

**Continuous Covariates**		**Non-stunted Mean value (SE)**	**Stunted** **Mean value (SE)**	**N**	
Age (months)		27 (0.4)	31 (0.4)	3593	-
Birth weight (kg)		3.4 (0.0)	3.3 (0.0)	3328	-
Preceding birth interval (months)		45 (0.7)	43 (0.7)	2586	-
** Categorical Covariates**		**Non-stunted (%)**	**Stunted (%)**	**N (%)**	OR*
Sex of child	Female	53.1	42.9	1768 (49.2)	1.00
	Male	46.9	57.1	1825 (50.8)	1.51
Exclusive breastfeeding	Yes	31.1	11.3	349 (25.2)	1.00
	No	68.9	88.7	1037 (74.8)	3.55
Diarrhoea in the last two weeks	No	11.3	15.4	462 (12.9)	1.00
	Yes	88.7	84.6	3131 (87.1)	1.42
Drug for intestinal parasite (in the last 2 weeks)	Yes	68.6	82.0	2646 (73.7)	1.00
	No	31.4	18.0	943 (26.3)	0.48
Minimum dietary diversity	Yes	21.7	21.2	316 (21.5)	1.00
	No	78.3	78.8	1152 (78.5)	1.03
Mother highest education	Secondary & higher	16.7	6.6	463 (12.9)	-
	Primary only	71.1	75.4	2615 (72.8)	
	No education	12.1	18.0	516 (14.4)	
Mother BMI	Underweight (BMI < 18.5)	4.0	6.1	154 (4.8)	-
	Normal	70.0	76.6	2323 (72.6)	
	Overweight & Obese (BMI ≥ 25)	26.0	17.3	724 (22.6)	
Sanitation	Improved	73.0	65.3	2494 (70.0)	1.00
	Non-improved	27.0	34.7	1067 (30.0)	1.43
Flooring	Good	27.1	12.6	769 (21.6)	1.00
	Poor	72.9	87.4	2796 (78.4)	2.58
Cooking fuels	Good/medium	18.9	9.2	540 (15.2)	1.00
	Poor	81.1	90.8	3023 (84.8)	2.30
Drinking water source	Improved	74.3	67.1	2551 (71.6)	1.00
	Non-improved	25.7	32.9	1014 (28.4)	1.42
Wealth index	Richest	21.3	9.1	599 (16.7)	-
	Richer	19.4	13.2	612 (17.0)	
	Middle	19.6	19.3	700 (19.5)	
	Poor	19.1	26.8	791 (22.0)	
	Poorest	20.6	31.6	891 (24.8)	
Type of residence	Urban	20.2	10.3	591 (16.4)	1.00
	Rural	79.8	89.7	3003 (83.6)	2.20

All variables were significant at *p*-value < 0.001, except for minimum dietary diversity (*p*-value = 0.840)

For the model inference, a Bayesian approach was used for estimating the posterior distribution of model parameters. The missing values in the covariates were considered as ignorable missing completely at random (MCAR) [16]. That is, we assume that there is no systematic difference in the missingness mechanism. Hence, we needed to specify an imputation model since we want include individuals with missing values in the model. We addressed this by using Bayesian imputation sub-models [17], where covariates with missing data were assumed as random variables rather than fixed. For the continuous covariates, a normal distribution likelihood was specified ${X}_{i}\sim N \left({\mu }_{Xi}, {\sigma }_{X}^{2}\right)$ with vague priors were assumed for the mean ${\mu }_{Xi}$ and variance ${\sigma }_{X}^{2}$. For the categorical covariates, we assumed a multinomial distribution ${Z}_{i}\sim MN(1,{\pi }_{ik})$, where ${\pi }_{ik}$ are the probabilities for the $k=1,..,K$ categories. Adapting the convention that for $k=1$ (the base category) the linear predictor ${\eta }_{Zi1}=0$, we model the probabilities as ${\pi }_{ik}=\mathrm{exp}({\eta }_{Zik})/\sum_{k}\mathrm{exp}({\eta }_{Zik})$. For $k=2,\dots ,K$, we assumed the linear predictor as ${\eta }_{Zik}\sim N \left({\mu }_{Zk}, {\sigma }_{Z}^{2}\right)$ with vague priors for the mean ${\mu }_{Zk}$ and variance ${\sigma }_{Z}^{2}$.

To complete the model, we assigned prior distributions for all unknown parameters. For the parameters of the continuous covariates, we assigned the normal priors ${\beta }_{p}\sim N(0,{\sigma }_{\beta }^{2})$ with uniform standard deviations ${\sigma }_{\beta }\sim U(\mathrm{0,10}$). For the categorical covariates, we set ${\gamma }_{1}=0$ as corner constraints for the reference categories and assigned the normal priors ${\gamma }_{l}\sim N(0,{\sigma }_{\gamma }^{2})$, $l=2,\dots ,J$, with uniform standard deviations ${\sigma }_{\gamma }\sim U(\mathrm{0,10}$). We modelled the spatially structured variation as zero-centered Gaussian random field. The joint density $s=\{{s}_{i},\dots ,{s}_{J}\}$ is a multivariate normal (MV) distribution $s\sim MV\left(0,\Sigma \right)$ defined by the isotropic exponential covariance function $\Sigma ={\sigma }_{s}^{2}\mathrm{exp}\left(-\phi d\right)$, where *d* is a $J\times J$ matrix of the distances between the clusters. We assigned a uniform standard deviation for the spatial variance parameter ${\sigma }_{s}\sim U(\mathrm{0,10}$) and the distance decay parameter (range) $\phi \sim U(\mathrm{0,1})$. We modelled the unstructured heterogeneity as normal exchangeable random intercepts ${u}_{j}\sim N(0,{\sigma }_{u}^{2})$ with uniform prior standard deviation ${\sigma }_{u}\sim U(\mathrm{0,10})$. Regarding the covariate imputation models for the continuous covariates, the assumed vague normal priors are ${\mu }_{X}\sim N(\mathrm{0,0.001})$ and uniform standard deviations for ${\sigma }_{X}\sim U(\mathrm{0,10})$. Similarly, we assumed vague normal priors for ${\mu }_{Zk}\sim N(\mathrm{0,0.001})$ and the uniform standard deviations for ${\sigma }_{Z}\sim U(\mathrm{0,10})$.

We fitted two separate models with similar strictures, except for the inclusion or exclusion of spatially structured random effects. Model 1 included only the spatially unstructured random effects, while model 2 included both the spatially structured and unstructured random effects. The models were implemented in WinBUGS 1.4 [18], using Markov Chain Monte Carlo (MCMC) simulation techniques. We run a chain of 40,000 iterations and discarded the first 20,000, obtaining a final sample of 20,000 for inference and summary statistics. Convergence was checked by visual inspection of the MCMC chains.

Residual predictions were made for the centroids of a 5 × 5 km grid designed within the study region. Assuming independence between the prediction locations, the prediction weights ${\lambda }_{oi}={\Sigma }_{oi}{\Sigma }_{ij}$ were estimated at each step of the MCMC iterations based on the covariances between the prediction and the observation locations ${\Sigma }_{oi}$ and the covariances between the observations ${\Sigma }_{ij}$. For any of the grids, the predicted residual at its centroid is a weighted average of the data, ${u}_{0}={\lambda }_{oi}{u}_{i}$. We used the *spatial.unipred* function in WinBUGS to undertake this task. An alternative function is the *spatial.pred* function based on conditional predictions which is rather extremely computationally intensive.

### Model validation and comparison

We used the cross-validatory posterior Bayesian probability values (B-*p-values*) and conditional predictive ordinates (CPO) to evaluate the predictive performance of the models. The CPO expresses the posterior probability of observing the outcome ${y}_{ij}$ when the model is fitted to all data except ${y}_{ij}$. Larger values imply a better fit of the model to ${y}_{ij}$, and very low CPO values suggest that ${y}_{ij}$ is an outlier with regard to the model being fitted [19]. The computation of B-*p-values* involves sampling replicates from the models ${y}_{ij}^{rep}\sim Bern\left({p}_{i}\right)$ and estimating the probabilities $\mathrm{Pr}\left({y}_{ij}^{rep}={y}_{ij}|y\right)$. The CPO is the equivalence of leave-one-out as cross validation. We calculated CPO for each observation as $\mathrm{Pr}\left({y}_{i}|{y}_{[ij]}\right)$, where ${y}_{[ij]}$ represents all data sets except ${y}_{ij}$. Observations with high B-*p-values* values are also indications of a good fit, while those with low values indicate a poor fit. For the comparison of models 1 and 2, we used the pseudo marginal likelihood (PsML) measure, also a pseudo Bayes factor. This is estimated as the product of the CPOs or the sum of their logged values [20]. A higher value of the PsML implies a better fit of a model to the observations.

### Prediction of spatial residual effects

The spatial residual effects of stunting were predicted using a 5 × 5 km grid for the whole surface of Rwanda. The 5 × 5 km grid surface was used to match previous published work on stunting prediction in Rwanda [9, 12]. The use of the 5 × 5 km grid takes into account the displacement of household clusters, conducted by the DHS to protect the privacy of respondents [21]. The spatial residuals were reported as odds ratios. Also, to understand the factors that could explain the predicted risk of stunting not accounted for by the geostatistical model, a visual comparison of the spatial pattern of the spatial residuals from this study was made with the results of a previous study by the authors on the spatial pattern of stunting in Rwanda [22].

## Results

### Study population

The descriptive characteristics of the study population are presented in Table 1. Stunted children on average were older (31 months) than non-stunted children (27 months). The child birth weight was 3.4 kg for non-stunted children and 3.3 kg for stunted children. The preceding birth interval was two months more for non-stunted children than for stunted children. In the study population, male children were 49.2% and female children were 50.8%; male children were more stunted (57.1%) compared to female children (42.9%). Exclusive breastfeeding was low in this study population, only 25.2% of children were exclusively breastfed in the last six months. Of all children, 12.9% had diarrhoea in the last two weeks. As many as 73.7% of the children had received drugs for intestinal parasites in the two weeks that preceded the survey. Children with a food consumption with minimum dietary diversity were only 21.5%. Mothers who had secondary education were 12.9% and those who did not have any education were 14.4%. Most mothers (72.6%) had a normal body mass index. Regarding household factors, 30% of the households relied on non-improved sanitation, 78.4% had poor flooring in their house, 84.8% used poor cooking fuels, and 28.4% used a non-improved source for drinking water. If we consider the wealth index, 24.8% of the households were in the category ‘poorest’, and 22.0% in the category ‘poor’. Most of the households (83.6%) live in rural areas, with only 16.4% living in an urban area. All factors, apart from minimum dietary diversity, were significantly different between stunted and non-stunted children.

**Fig. 2:**
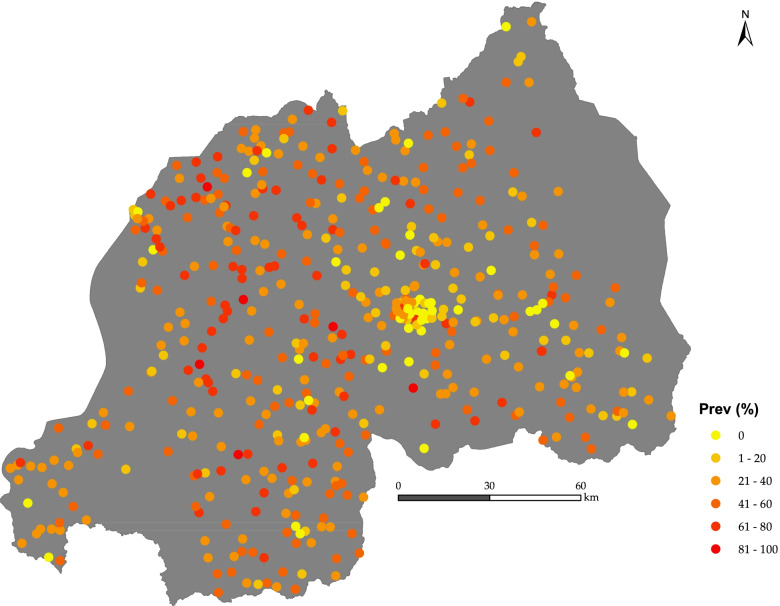
Stunting prevalence at household cluster level in Rwanda (source: DHS, 2015). Map created using ArcGIS Desktop version 10.6, licensed (https://www.esri.com/)

Figure 2 indicates the stunting prevalence of children in Rwanda at the household cluster level. At the household cluster level, the prevalence varies from 0 to 100%. High stunting prevalence was predominantly found in the Western and Southern parts of the country. Kigali province has more household clusters with zero stunting prevalence.

### Model fit and comparison

The B-*p-values* and CPO values ranged from 0.12 to 0.96 and 0.11 to 0.95 for model 1 respectively. For model 2, the B-*p-values* and CPO values respectively ranged from 0.12 to 0.96 and from 0.11 to 0.95. These values indicate a good fit for both models since no extreme low values were observed. Dividing either by their maximum to scale to a maximum of one, the lowest values were approximately 0.12 for both models, far greater than the limit of 0.001 suggested by Weiss (1994). The PsML for model 1 was -2050.1 and that of model 2 was -2035.7, indicating that model 2 is superior to model 1.

### Risk factors of stunting

The posterior means odds ratios and the 95% confidence intervals for the odds ratio of the Bayesian spatial model are shown in Table 2. The geostatistical model indicated that age of child, child’s sex, birthweight, exclusive breastfeeding, occurrence of diarrhoea in the last two weeks, BMI of the mother, and education level of the mother were significantly associated with stunting. The type of flooring material of the house, type of water source used, and wealth index category also predicted stunting significantly.

**Table 2:** Risk factors for childhood stunting in Rwanda, 2015 from the binary logistic Bayesian geostatistical model

**Covariates**		**OR**	**95% CI**
Age (months)^a^		1.015	(1.010, 1.024)
Birthweight (kg)^a^		0.961	(0.950, 0.973)
Preceding birth interval (months)^a^		1.000	(0.990, 1.004)
Sex of child	Female	1.000	
	Male	1.320	(1.161, 1.473)
Exclusive breastfeeding	Yes	1.000	
	No	1.241	(1.045, 1.452)
Diarrhoea in the last two weeks	No	1.000	
	Yes	1.181	(1.021, 1.373)
Drug for intestinal parasite (in the last 2 weeks)	Yes	1.000	
	No	0.762	(0.671, 0.874)
Minimum dietary diversity	Yes	1.000	
	No	0.931	(0.791, 1.084)
Mother highest education	Secondary & higher	1.000	
	Primary	1.142	(0.991, 1.312)
	No education	1.154	(0.991, 1.364)
Mother BMI	Underweight	1.000	
	Normal	1.011	(0.886, 1.153)
	Overweight & Obese	0.823	(0.711, 0.953)
Sanitation	Improved	1.000	
	Non-improved	1.061	(0.943, 1.214)
Flooring	Good	1.000	
	Poor	1.224	(1.062, 1.414)
Cooking fuels	Good/medium	1.000	
	Poor	1.063	(0.883, 1.243)
Drinking Water source	Improved	1.000	
	Non-improved	1.134	(1.001, 1.272))
Wealth index groups	Richest	1.000	
	Richer	0.893	(0.763, 1.034)
	Middle	1.012	(0.875, 1.171)
	Poorer	1.151	(0.991, 1.334)
	Poorest	1.240	(1.074, 1.424)
Type of residence	Urban	1.000	
	Rural	1.143	(0.992, 1.354)

^a^The continuous variables were centered around the mean. *OR* odds ratios

Older children have a slightly higher risk of stunting. Children with a higher birth weight had a lower stunting risk (OR = 0.96, 95% CI: 0.95, 0.97). Male children had an increased risk of stunting compared to female children (OR = 1.32, 95% CI: 1.16, 1.47). Exclusive breastfeeding was protective against stunting. The odds of stunting were 24% higher in children who were not exclusively breastfed compared to children who were exclusively breastfed (OR = 1.24, 95% CI: 1.04, 1.45). Similarly, the odds of stunting among children who had diarrhoea in the last two weeks preceding the survey was higher than those who did not have diarrhoea (OR = 1.18, 95% CI: 1.02, 1.37). The odds of stunting were 15% higher among children whose mothers had no or only primary education, compared to children whose mothers had secondary and higher education level (OR = 1.15, 95% CI: 0.99, 1.36). Poor flooring of the inhabited house also significantly increased the risk of stunting in children (OR = 1.22, 95% CI: 1.06, 1.41). As could be expected, children whose household is categorised as ‘poorest’ also had an elevated risk of stunting (OR = 1.24, 95% CI: 1.07, 1.42). Use of a non-improved water source also increased the risk of stunting by 13% compared to households that do have access to an improved water source (OR = 1.13, 95% CI: 1.00, 1.27). The type of residence also was significantly associated with stunting, with households in rural areas having a higher risk of stunting compared to households in urban areas (OR = 1.14, 95% CI: 0.99, 1.35). Although some covariates such as the preceding birth interval, type of cooking fuels, and sanitation were not significantly associated with stunting in the spatial model, the direction of their relationship with stunting was as one would expect. In the univariate analysis (Table 1) these covariates were significantly associated with stunting.

### Spatial residual effects prediction

**Fig. 3:**
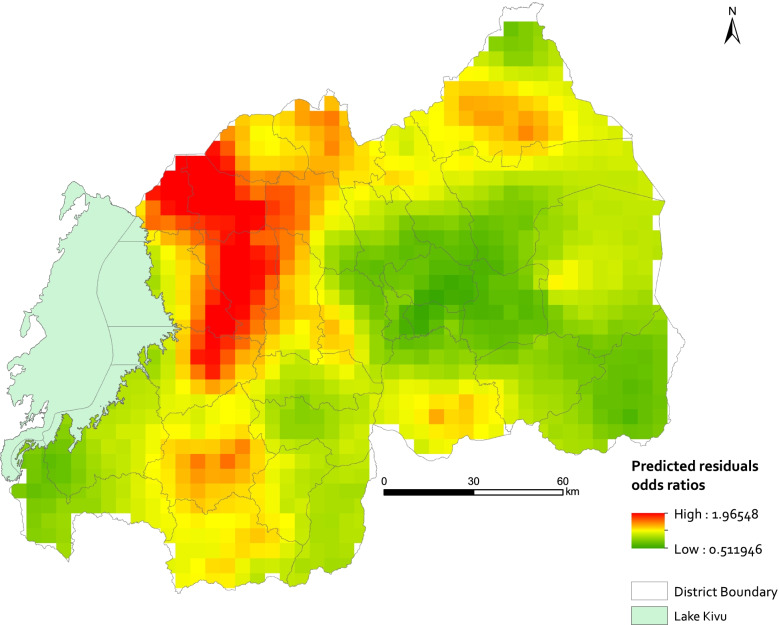
Spatial residual effects of stunting expressed as odds ratios on a 5 × 5 km grid resolution based on geo-located household cluster-level data. Map created using ArcGIS Desktop, version 10.6, licensed (https://www.esri.com)

**Fig. 4:**
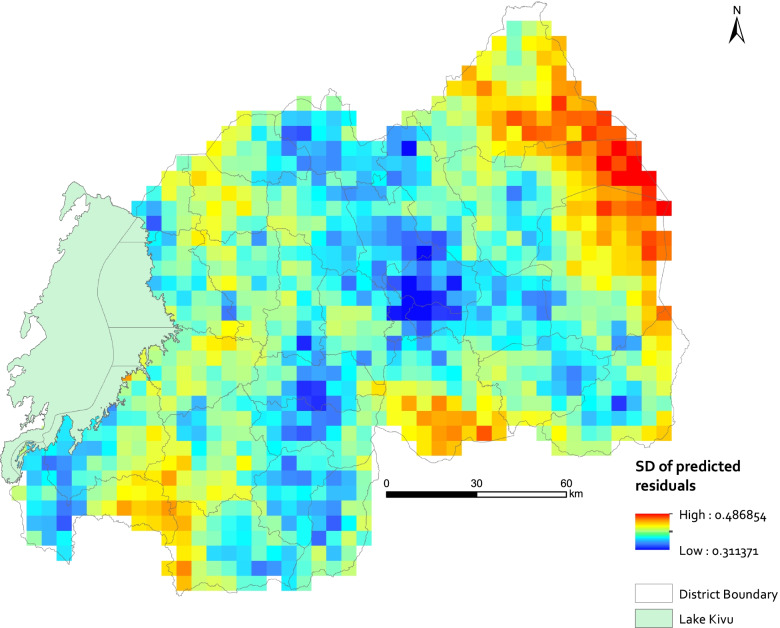
Uncertainty map for the posterior spatial residual effects displayed as standard deviation (SD) on 5 × 5 km grid resolution. Map created using ArcGIS Desktop version 10.6, licensed (https://www.esri.com/)

Figure 3 shows the spatial residual effects for stunting after accounting for the covariates in the binary logistic spatial model, and Fig. 4 displays the corresponding prediction uncertainty. In Fig. 2, areas with higher odds ratios have increased risk of stunting, and areas with lower odds ratios have decreased risk of stunting in children less than five years, again after accounting for the covariates in the geostatistical model. The central area of Rwanda that includes Kigali province, and the Eastern part show unexplained lower risk of stunting, while the Western, Northern and Southern regions show unexplained higher risk of stunting.

## Discussion

Our study provides evidence of the spatial variation of stunting in Rwanda and the risk of stunting that is still persistent after individual, maternal, socio-economic and dietary factors were taken into account. In the spatial model, child characteristics such as child age, child sex, birthweight, exclusive breastfeeding and having had diarrhoea in two weeks that preceded the survey were important predictors of stunting. Previous research also showed that male children are more likely to be stunted than female children [23–25]. The odds of stunting increased as the age of children increased. However, the odds were not higher than previously identified in other studies [26, 27], probably because age was considered as a continuous variable in the model. Consideration of age as a categorical variable would classify the risk of stunting per age group, and this risk has been shown to be higher in older children than in younger children [28, 29]. A higher birth weight of children was associated with slightly lower odds of stunting. Children who are born with a low birth weight (< 2.5 kg) have a higher risk of stunting in the first 1000 days of their life [30]. In this study, non-exclusive breastfeeding in the previous six months was associated with the second highest odds of stunting after child’s sex. The protective effect of exclusive breastfeeding on child stunting is a well-established evidence [5, 31]. Thus, the need for mothers to exclusively breastfeed their children in the first six months of life cannot be overemphasized. Children who had diarrhoea in the previous two weeks had increased odds of stunting compared to children who did not. This is consistent with the available evidence on the negative effect of diarrhoea on the linear growth of children [32]. Early childhood enteric infections have been shown to contribute to malnutrition, which in turn leads to increased vulnerability to infections [33, 34]. Micronutrients such as vitamin A and zinc deficiencies have been linked to the disruption of intestinal barrier and absorption functions [34]. In Rwanda, although most younger children are supplemented with vitamin A every six months [10], the dietary intake of these crucial micronutrients is generally still below nutrient requirements especially for dietary zinc intake [29, 35]. Thus, to break this vicious cycle, proper sanitation facilities, coupled with balanced nutrition, especially in impoverished regions are still urgently needed. Unsurprisingly, households with access to a non-improved water source had higher odds of stunting compared to households with access to an improved water source. The same pattern was observed for households with poor flooring and households located in rural areas. These results confirm the impact socio-economic conditions on increasing or reducing the risk of stunting. The consistent high levels of stunting found in children living in rural areas compared to their counterparts living in urban areas clearly illustrates this [10, 25].

The maternal factors considered in the model, mother BMI and education level, were both significantly associated with stunting. Children of overweight mothers had less risk of becoming stunted compared to children from underweight mothers. Also, having a mother with no or only primary education increased the risk of stunting compared to having a mother with secondary or higher education. The predicted association of mother’s BMI and education with stunting is consistent with the findings of previous studies [36–38], and emphasizes the importance of mother’s health and their knowledge on the nutritional wellbeing of children. Although children of overweight and obese women were at low risk of stunting in this study, maternal obesity has been shown to increase the risk of childhood obesity, which can continue into adolescence and adulthood [38]. Children whose household was in the poorest category of the wealth index had the highest odds of stunting compared to children from households in the richest category. The poorest category was the only statistically significant category, compared to poorer, middle and richer categories. [39] reported a similar positive association where living in absolute poverty was associated with poor child development.

The spatial heterogeneity of predicted residuals observed in Rwanda (Fig. 3) suggests that unobserved factors, not accounted for by the covariates in the geostatistical model, contribute to the geographical disparities in stunting outcomes. The predicted spatial residual effects depict a spatial pattern of the risk of stunting which is pronounced mainly in the Western region, followed by the Northern and Southern regions of Rwanda. As stunting is multifactorial, the factors contributing to stunting require further investigation. Although our model included not only child related factors but also dietary covariates, other child and non-child related factors might be contributing to the unexplained variance. We believe among the factors non-accounted for in the model, that mycotoxins exposure might very well be an important factor [40]. This is because there is a strong and well-established evidence of the negative effect of mycotoxins exposure on the growth of children. Other research has shown that mycotoxins exposure, especially during the complementary feeding period, is associated with stunting in children [40–45]. The lack of geographically explicit mycotoxins data in Rwanda has been highlighted by the authors before as a barrier in further analysing this potential relation [22].

Preceding research by Uwiringiyimana, Veldkamp [22] on the spatial pattern of stunting in Rwanda developed a proxy measure of aflatoxins exposure. In this study, we again used this proxy variable of mycotoxins exposure but now to map and inspect if a relationship can be identified with the unexplained variance in stunting as predicted by our geostatistical model. The proxy variable for mycotoxins developed by [22] classified household clusters as being at high or low risk of mycotoxins exposure depending on whether clusters were served by an urban market, a rural market or neither served by an urban nor a rural market. An urban market was considered to be at the start of the food supply chain, while a rural market was considered to be at the lower end of the food supply chain of complementary flours. Household clusters served by an urban market were considered to have a lower risk of exposure to contaminated complementary flours than clusters served by rural markets at the lower end of the food supply chain [22]. Household clusters not served by an urban nor by a rural market, *i.e.* the most remote areas, were considered to have the highest risk of exposure to contaminated flours.

**Fig. 5:**
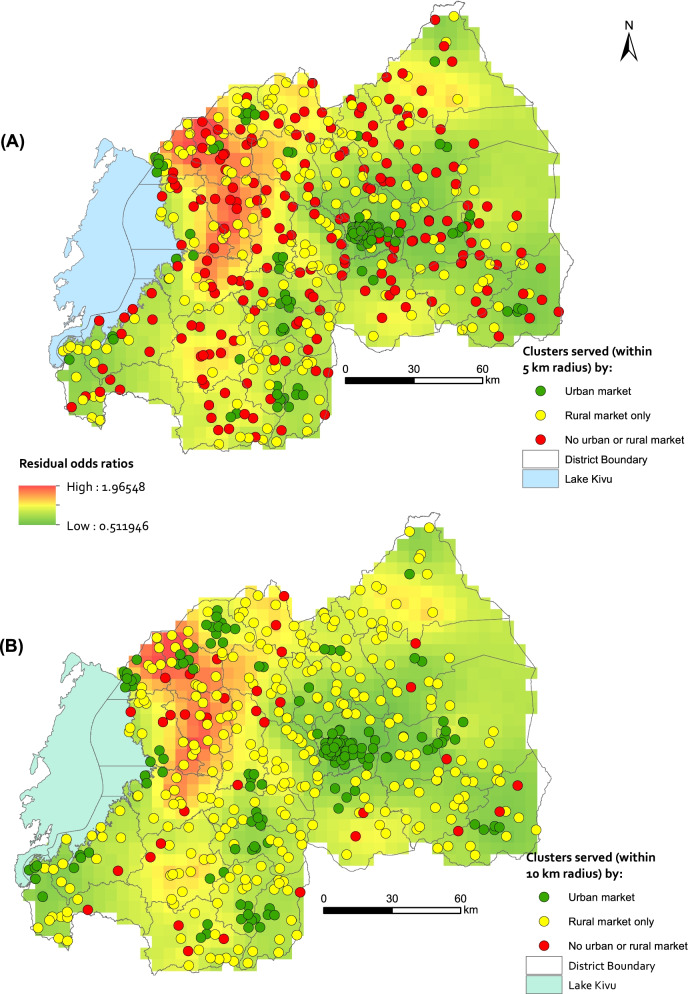
Spatial residual effects map overlaid with the spatial distribution of household clusters that are served by either an urban market, a rural market only, and neither an urban nor a rural market within a 5 km (A) and 10 km (B) radius of the household cluster. Maps created using ArcGIS Desktop version 10.6, licensed (https://www.esri.com/)

Figure 5 shows the spatial residual effects map together with the spatial distribution of household clusters that are served by an urban market, a rural market only, and neither an urban nor a rural market within a 5 km (A) and 10 km (B) radius of the household cluster. For both maps, especially panel B, the green zones representing lower risk of stunting are associated with household clusters that are served by urban markets. This matches with our hypothesis that household clusters served by an urban market have a lower risk of exposure to mycotoxins. In other words, household clusters served by an urban market are likely to have access to better quality of food. The correlation between the residual odds ratios, sampled at each household cluster location, and the three classes of household clusters was significant for both panel A (*r* = 0.232, *p*-value < 0.01) and panel B (*r* = 0.279, *p*-value < 0.01). Also, a pattern can be observed in the Western and Southern provinces where a high unexplained risk of stunting coincides with household clusters that are not served by an urban and not by a rural market (Fig. 5A), and with household clusters that are served by mostly rural markets (Fig. 5B). On the other hand, the Eastern region, which also features clusters with no access to an urban or a rural market (Fig. 5A), depicts a lower risk of stunting. We expect that there is an interplay of factors both in the regions with high unexplained risk of stunting or regions with low unexplained risk of stunting, children in the high-risk regions might be already at a disadvantage, in such a way that the addition of mycotoxins contamination more drastically affects them compared to children whose only risk would be the exposure to mycotoxins. We underline that this comparison takes into account the limitations of using the proxy variable of exposure to mycotoxins as discussed in [22].

Elevation is another factor that could explain the residual variability in stunting as it has been shown to be associated with stunting in children [46, 47]. Although not considered in our model, the regions with a high unexplained risk of stunting coincide with regions with high altitude (see Fig. 3). Previous research spatially predicted stunting on a national level in Africa and Rwanda [9, 12]. However, the prediction of stunting was done using non-child related covariates such as surface temperature, night time lights and elevation. Our study strength lies in using child-related factors that are known to have a causal relationship with stunting in our geostatistical model, to study the risk factors of stunting in Rwanda, and predict the spatial residuals effects of stunting. Predicting the spatial residual effects by incorporating individual and household related factors has been done previously in other studies, to better understand the spatial distribution of *Schistosoma mansoni* infection in Ivory Coast [48] and of malaria in Malawi [49]. All the variables included in the spatial model, except for the minimum dietary diversity, were strong predictors of stunting in children. Although the association of the minimum dietary diversity with stunting was not significant in the univariate analysis, we kept the covariate in the spatial model because of its known effect on stunting in children. More generally, applying Bayesian geostatistical modelling to our data had different advantages. First we could apply prior knowledge in the model using published sources [12]. Second, using covariates strongly associated with stunting, the spatial residual effects of stunting that display the unexplained risk of stunting in Rwanda were predicted. Third, normal regression methods usually applied to study the risk factors of stunting do not take into account the spatial heterogeneity and spatial dependence that exist in stunting. Our model used the geographical locations of household clusters to estimate the spatial dependency in childhood stunting in Rwanda.

Our study also had some limitations. First, because the covariates such as exclusive breastfeeding and minimum dietary diversity are measured by DHS only for children < 2 years, this introduced missing values in the data for all children aged ≥ 2 years. Consequently, the small sample of children for which dietary data were available, might have led to the lack of association between minimum dietary diversity and stunting. Second, although our study is the first to apply geostatistical modelling to predict the unexplained risk of stunting in Rwanda, our model did not take into account environmental factors that might influence stunting. Thus, further research on modelling the unexplained risk of stunting can further study the underlying factors that determine the spatial pattern of the higher and lower odds of the spatial residuals effects observed in this study. The model can be strengthened by incorporating non-child related factors that could shed light on the unexplained odds ratios of stunting. Also, for future research, the extent of the negative effect of mycotoxins exposure on growing children urgently needs to be evaluated. Studies determining the extent of mycotoxins contamination along the food supply chain in Rwanda, by taking into account the spatial variation in mycotoxins, can provide the needed data to better examine this relationship. Such research would serve to inform policy on the extent of mycotoxins contamination in the food supply chain and provide spatially informed evidence of the effect of mycotoxins exposure on the spatial pattern of stunting in Rwanda.

## Conclusion

Stunting in Rwanda varies spatially at a much finer geographical scale than the district level which is the usual spatial unit to present levels of stunting in Rwanda. Our study applied an advanced geostatistical method to study the risk factors of stunting in Rwanda and predict the unexplained odds of stunting in the study population. Although stunting is multifactorial, studies that take into account spatial dependency of stunting are necessary to obtain improved understanding of its drivers on a local scale. Using Bayesian geostatistical modelling offers the opportunity to study the spatial pattern of stunting by taking into account its spatial dependency, and by revealing residual risk of stunting unexplained by the model. The identification of areas with a high spatial residual odds ratio implies that further research is needed to identify currently unknown but spatially explicit factors associated with high stunting risk.

## Data Availability

The anthropometric, socioeconomic and demographic data that support the findings of this study are publicly available on request from the Demographic and Health Survey (DHS), https://dhsprogram.com/data/available-datasets.cfm

## Abbreviations

BMI: Body mass index
B-*p-values*: Bayesian probability values
CI: Confidence interval
CPO: Conditional predictive ordinates
DHS: Demographic health survey
MCMC: Markov chain Monte Carlo simulation
OR: Odds ratio
PsML: Pseudo marginal likelihood
SD: Standard deviation
SE: Standard error
SPSS: Statistical package for the social sciences
WHO: World Health Organization
